# Implicitly learning when to be ready: From instances to categories

**DOI:** 10.3758/s13423-021-02004-w

**Published:** 2021-10-28

**Authors:** Wouter Kruijne, Riccardo M. Galli, Sander A. Los

**Affiliations:** 1 Department of Experimental Psychology, Faculty of Behavioral and Social Sciences, University of Groningen, Grote Kruisstraat 2/1, 9712 TS Groningen, Netherlands; 2 UKE Hamburg, Hamburg, Germany; 3 VU Amsterdam, Amsterdam, Netherlands

**Keywords:** Temporal preparation, Long-term memory, Prediction, Generalization, Time-course analysis

## Abstract

There is growing appreciation for the role of long-term memory in guiding temporal preparation in speeded reaction time tasks. In experiments with variable foreperiods between a warning stimulus (S1) and a target stimulus (S2), preparation is affected by foreperiod distributions experienced in the past, long after the distribution has changed. These effects from memory can shape preparation largely implicitly, outside of participants’ awareness. Recent studies have demonstrated the associative nature of memory-guided preparation. When distinct S1s predict different foreperiods, they can trigger differential preparation accordingly. Here, we propose that memory-guided preparation allows for another key feature of learning: the ability to generalize across acquired associations and apply them to novel situations. Participants completed a variable foreperiod task where S1 was a unique image of either a face or a scene on each trial. Images of either category were paired with different distributions with predominantly shorter versus predominantly longer foreperiods. Participants displayed differential preparation to never-before seen images of either category, without being aware of the predictive nature of these categories. They continued doing so in a subsequent Transfer phase, after they had been informed that these contingencies no longer held. A novel rolling regression analysis revealed at a fine timescale how category-guided preparation gradually developed throughout the task, and that explicit information about these contingencies only briefly disrupted memory-guided preparation. These results offer new insights into temporal preparation as the product of a largely implicit process governed by associative learning from past experiences.

**Supplementary Information:**

The online version contains supplementary material available at 10.3758/s13423-021-02004-w.

It is a nearly universal fact of life that it is good to be prepared. Anticipating upcoming stimuli and events involves a cognitive process that allows us to react faster and more accurately to them. Optimal preparation involves not only predicting *what* action to take, but also *when* to take it. The benefits of *temporal preparation* range from carrying a pleasant conversation without interrupting our partner, to being able to avoid catastrophic incidents when navigating busy streets by car. Like most skills, making well-timed actions takes practice to master. The basics might be easy enough to acquire, but it can take a lifetime of learning to tell perfectly timed jokes as a successful comedian, or to obtain the split-second response times of a professional race car driver.

The neurocognitive underpinnings of temporal preparation are commonly studied using the ‘foreperiod paradigm’ (Woodrow, 1914; Niemi & Näätänen, 1981). Participants observe a warning stimulus (S1) which signals an upcoming target stimulus (S2) after an interval called the foreperiod (FP; typically in the range of 250–5000ms). Reaction times (RTs) of responses to the S2 are then used to investigate when participants are optimally prepared. In experiments with a variable FP, a typical result is that RTs decrease for trials with longer FPs, giving rise to a characteristic downwards-sloping curve that reaches an asymptote for longer FPs (Niemi & Näätänen, 1981). Interestingly, the shape of this RT-FP curve is strongly affected by the context in which trials are presented, demonstrating how preparation is a flexible process and modulated by past experiences. For example, participants generally respond rather slowly on short-FP trials when the FP on the preceding trial was longer, leading to a steep RT-FP curve (Los & Heslenfeld, 2005; Los & Agter, 2005; Steinborn & Langner, 2012). Conversely, if the FP on the preceding trial was short the RT-FP curve is flatter, with fast responses at all FPs. In similar fashion, preparation is modulated by the distribution of FPs throughout a block: when short FP-trials are most prevalent, as with an ‘exponential distribution’ (cf. Fig. 1C), participants respond relatively fast on both short- and long- FP trials. In blocks with an ‘anti-exponential distribution’, with primarily longer FPs, responses on rare short-FP trials tend to be very slow (Joubert & Baumeister, 1970; Zahn & Rosenthal, 1966; Niemi & Näätänen, 1981).

**Fig. 1:**
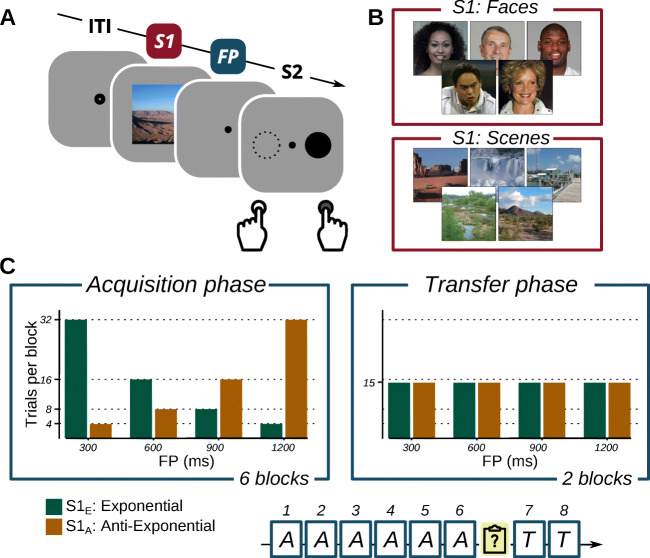
Trial sequence, stimuli, and block design of the experiment. *Note*. **A** Trial sequence: after a variable ITI, S1 was presented (200ms), followed by a variable FP which was terminated by S2. S2 was a circle on the left or right side prompting a left- or right handed response. **B** Example S1 stimuli. Unique stimuli were drawn on each trial from a set of faces or scenes. **C** During the *Acquisition* phase (6 blocks), images of either category were paired with an exponential or anti-exponential foreperiod distribution. Subsequently, participants completed a questionnaire and were informed that this contingency no longer held. During the following Transfer phase (2 blocks), both S1s were paired with the same, uniform FP distribution

Influential theories of temporal preparation have proposed that these contextual modulations are a consequence of participants’ temporal expectations, which are derived from their knowledge of the current FP distribution (Trillenberg, Verleger, Wascher, Wauschkuhn, & Wessel, 2000; Vangkilde et al.,, 2013; Nobre, Correa, & Coull, 2007; Janssen & Shadlen, 2005; Grabenhorst, Michalareas, Maloney, & Poeppel, 2019). While these theories offer a mathematical description of preparation effects, they typically do not define how the underlying distributions are learned, maintained, and updated (though see Meindertsma, Kloosterman, Engel, Wagenmakers, & Donner, 2018; Visalli, Capizzi, Ambrosini, Mazzonetto, & Vallesi, 2019, for recent proposals), nor do they specify how such expectations are translated into faster or slower responses. The Multiple Trace Theory of Temporal Preparation (MTP; Los, Kruijne, & Meeter, 2014; Salet, Kruijne, Van Rijn, Los, & Meeter, under review) seeks to address these issues by presenting a process model of temporal preparation at a more mechanistic level of abstraction. MTP proposes that preparation is guided by Hebbian learning between a dynamic neural representation of time that evolves during the foreperiod (cf. Howard & Eichenbaum, 2013; Machado 1997; Shankar & Howard, 2011), and motor processes that govern the inhibition and activation of prepotent responses (cf. Davranche, Tandonnet, Burle, Meynier, Vidal, & Hasbroucq, 2007; Duque & Ivry, 2009; Los, 2013; Näätänen, 1971; Narayanan & Laubach, 2006). These associations are formed on individual trials as episodic memory traces. On subsequent trials, the associative retrieval of these traces affects the balance of motor activation and inhibition throughout the foreperiod, which in turn yields faster or slower responses.

MTP can be viewed as an ‘instance theory’ (Logan, 1990; 2002; Schneider & Shiffrin, 1977). A defining assumption of instance theories is that cognitive performance is automatically and implicitly modulated by the associations formed on individual past trials. Instance theories have been applied to account for findings on reinforcement learning (Doll et al.,, 2015; Bornstein, Khaw, Shohamy, & Daw, 2017), temporal reproduction (Taatgen & van Rijn, 2011), visual attention (Chun & Jiang, 1998; Kruijne & Meeter, 2015; Turk-Browne, Jungé, & Scholl, 2005), stimulus-response bindings (Henson, Eckstein, Waszak, Frings, & Horner, 2014; Horner & Henson, 2009; Longman, Milton, Wills, & Verbruggen, 2018) and task switching (Waszak, Hommel, & Allport, 2003). MTP adopts this perspective to account for temporal preparation phenomena.

Conform other instance theories, MTP posits that associative long-term memory has a key role in guiding temporal preparation. Two studies have directly investigated such guidance by associations. In one of these (Cravo, Rohenkohl, Santos, & Nobre, 2017), participants explicitly learned to associate different S1s (photographs of scenes) with either a short or long FP. Behavioral and electrophysiological results indicated that these associations modulated preparation on future trials with the same S1s. Furthermore, the memory strength of these associations predicted long-lasting differences in preparatory behavior in subsequent blocks. Another study (Los, Nieuwenstein, Bouharab, Stephens, Meeter, & Kruijne, 2021) highlighted how associations may modulate preparation implicitly, outside of voluntary control. Experiments consisted of two phases: an ‘Acquisition phase’ where two different S1s (e.g., a tone or flash) were associated with either an exponential or anti-exponential FP distribution, followed by a ‘Transfer phase’ where FPs were distributed uniformly for either S1 (cf. Fig. 1C). Results showed that throughout the Acquisition phase participants adapted preparation to the distribution predicted by the S1, even though follow-up questionnaires indicated they had been unaware of the contingency. Critically, differential preparation persisted far into the Transfer phase, even though participants were instructed that the contingency no longer held in the upcoming blocks (see Los, Kruijne, & Meeter, 2017; Mattiesing, Kruijne, Meeter, & Los, 2017, for similar results).

While both Cravo et al., (2017) and Los et al., (2021) demonstrate that long-term memory associations can guide preparation, there are marked differences between these paradigms in how associations were operationalized. The results of Cravo et al., (2017) resemble ‘declarative’ memory, as participants seemingly used explicit knowledge of each S1-FP association. The results of Los et al., (2021), however, are more akin to ‘procedural’ memory, where repeated practice with either S1 results in a statistical ‘rule’ guiding preparation without explicit knowledge. Such differences impede strong conclusions regarding the exact nature of associations that modulate preparation, and whether declarative knowledge may be imperative to learn from unique individual instances.

Here, we aim to further elucidate the nature of the long-term associations that guide preparation. In particular, we investigate whether individual memories of unique trials can give rise to a statistical rule that generalizes to novel stimuli. To this end, participants were presented with unique S1-images from one of two categories (faces and scenes). Participants were not informed that these categories were associated with an exponential or anti-exponential FP-distribution during Acquisition. We assessed whether this association would yield differential preparation to never-before seen images of either category. Furthermore, we assessed whether differential preparation would persist into a Transfer phase where both categories were paired with uniform FP distributions. Using a novel rolling regression analysis, we mapped the development of differential preparation at a fine-grained time scale, and offer new insights into how these long-term associations interact with countermanding instructions.

## Methods

Data and analysis scripts are available on OSF (https://osf.io/s7xp6/), alongside supplemental information. The experiment was not preregistered.

### Participants

Participants were recruited through the participant pool of the Vrije Universiteit Amsterdam, and completed the experiment in exchange for either course credit or monetary compensation. The experimental procedure was approved by the Ethical committee of the Faculty of Behavioral and Movement Sciences. Participants were treated in accordance with the guidelines of the Helsinki declaration, and gave informed consent before participating. The experiment was completed by 50 participants (power analysis reported below). One participant was excluded from further analysis as her mean RT on correct trials was more than 2.5SD away from that of the sample. Remaining participants’ accuracies were all higher than the predefined cut-off value (95%), so no other datasets were discarded. The final sample included 49 participants (37 female; ages 18–30; Mean age 23.3).

### Task and stimuli

Participants were seated in a dimly lit cubicle at 70cm viewing distance, asserted by a chin rest, from a $22^{\prime \prime }$ monitor with 120Hz refresh rate at 1280 x 768 px. The experiment was designed and run using OpenSesame version 3.3 (Mathôt, Schreij, & Theeuwes, 2012). Participants were informed that on each trial they would be presented with a warning stimulus (a photograph, the S1), which would be followed by a target stimulus (a black circle, the S2) presented either on the left or right of the screen. They were instructed to maintain fixation at the center of the screen, and to respond as fast as possible to the location of the S2, by pressing ‘Z’ or ‘M’ on a keyboard for ‘left’ or ‘right’ targets respectively. They were told to be as fast as possible, while maintaining a high accuracy.

All stimuli except for S1 were black and were presented on a gray background (Fig. 1A). During the intertrial interval (ITI, randomly sampled between 750–1500ms), an open fixation dot (0.55^∘^ diameter) was presented in the center of the screen. A trial started with the presentation of the warning stimulus (S1): a square, color photograph of a face or a scene (sides of 8.66^∘^) presented in the center of the screen for 200ms. What followed was a variable FP during which only a small fixation dot (0.28^∘^ diameter) was presented, followed by the presentation of the target stimulus (S2). S2 was a filled black circle (1.40^∘^ diameter) presented to the left or right of fixation, at 5.22^∘^ eccentricity. S2 stayed on screen until the participant responded.

The images of Faces and Scenes that were used as S1s were taken from two databases. Faces were drawn from the ‘Labeled Faces in the Wild’ database (Huang, Mattar, Berg, & Learned-Miller, 2008; Huang & Learned-Miller, 2014; Learned-Miller, Huang, RoyChowdhury, Li, & Hua, 2016). A subset of this dataset was created, selecting images by discarding duplicate identities, images with poor quality or occlusion of the face, and images of well-known people.^1^ Scene images were selected from the SUN database (Xiao, Hays, Ehinger, Oliva, & Torralba, 2010), selecting only images labeled as ‘outdoors’ and discarding images of poor quality or with prevalent human faces in them. A list of selected images (515 face-images and 482 scene-images) is available as online supplemental information on OSF. Each image was cropped to a square image and scaled to 250 × 250px. For face images, the face-detection functions of OpenCV were used to center the cropped image on the face. From the resulting set, a unique image was selected on each trial to be used as S1.

### Design and procedure

The FP on each trial was defined as the stimulus-onset asynchrony between S1 and S2 and was either 300, 600, 900, or 1200 ms. Each S1 category (faces or scenes) was associated with a different discrete distribution of FPs during the Acquisition phase (Fig. 1C). That is, one S1 type (*S*1_*E*_) was associated with an *exponential* distribution, with 32, 16, 8 and 4 trials per block for each FP, respectively. The other S1 type (*S*1_*A*_) was associated with the inverse, *anti-exponential* distribution of FPs. The pairing of the two S1 types to the two image categories was fully counterbalanced across participants (see also Table S1 on OSF). Crucially, the contingency was only defined at the level of the S1 *category*: that is, individual S1 images were used only once throughout the experiment for each participant. The location of the S2, which defined the response, was fully balanced across the two S1 types within each block.

The Acquisition phase was followed by a Transfer phase, during which both *S*1_*E*_ and *S*1_*A*_ were associated with a uniform distribution, with 15 trials per FP for each S1 type. Blocks in either phase thus comprised 120 trials in total, with randomly intermixed FPs and S1 types. Because the S1 was unique on each trial, we expected that differential preparation would take longer to develop than in our earlier work Los et al., (2021). Therefore we used a relatively long Acquisition phase of six blocks, followed by a shorter Transfer phase of two blocks.

We included a brief questionnaire at the end of the Acquisition phase, to test whether participants might have developed explicit knowledge of the contingency, and could have used that to guide differential preparation. To this end, the experiment was interrupted and participants answered an open question whether, up to that point, they had been aware of any regularities in the experiment. Subsequently, they were asked a multiple-choice (MC) question whether (1) Faces were more often followed by short FPs and scenes by long FPs; (2) vice versa; (3) they did not know. Finally, they were fully informed about the predictive nature of the image categories that had applied up to that point. This was done for full disclosure, and to ensure that participants were all aware of the past contingency regardless of whether they had noticed it or not. Next, it was stressed that this contingency would no longer hold in the final two Transfer blocks, and that short and long FPs would be equally likely after either S1 type. The list of responses to both questions is given as Supplemental Table S1 on OSF. After this ‘intervention’, the experiment continued with the Transfer phase, starting with Block 7.

### Statistical analyses

We discarded trials with incorrect responses, as well as trials with a log RT more than 3SD away from each participants’ mean (trials discarded per participant: M = 0.8%, SD = 1.5%). The primary approach for analyses of the remaining RTs was hypothesis-driven model comparison using Linear Mixed effects Models (LMMs; Baayen, Davidson, & Bates, 2008; Baayen & Milin, 2010). We anticipated preparation to manifest as a downwards sloping RT-FP curve, and that differential preparation for different S1 types would manifest as an interaction effect, modulating the slope to be steeper with *S*1_*A*_ and flatter with *S*1_*E*_ (cf. Coull, Frith, Büchel, & Nobre, 2000; Cravo et al., 2017; Los et al., 2017, 2021). To this end, our primary analyses compared models where RT was predicted by FP only versus a more complex model where S1 type (*S*1_*A*_/*S*1_*E*_) interacted with FP. All model comparisons were based on BIC scores converted to estimated Bayes Factors (BFs, following Wagenmakers, 2007). Wherever we report evidence for the inclusion of a term, we will report Δ*BIC* > 0 and *BF* > 1; evidence for exclusion is expressed as Δ*BIC* < 0 and 1/*BF* > 1.

Following Los et al., (2021), FP was coded as a continuous linear predictor. This is a simplification of the typically asymptotic RT-FP curve, but allowed us to express preparation effects in a single unambiguous model coefficient. To overcome the typical skew in RT distributions, models were fit on inverse RT (1/*RT*) as the dependent variable (motivated by the guidelines of Baayen et al., 2010; Lo & Andrews, 2015). Residuals of models with this structure gave no indication of any systematic misfits, suggesting that it accurately captures the RT-FP curve. In each model, hierarchical variance across participants in the intercept and the slope of the RT-FP curve were captured as a random intercept with a correlated random effects-term for FP. No other random effects were supported through model comparisons, and were therefore not considered.

In a first analysis we evaluated the effect of S1 type interacting with FP, and assessed whether this effect might have varied across the Acquisition versus the Transfer phase. As these analyses indicated that S1 type modulated preparation in both phases, we subsequently sought to identify how these effects developed throughout both phases. In earlier work, we did this by analyzing experimental blocks separately. However, a block-wise analysis reduces experimental power considerably, and by treating blocks as independent observations this approach ignores the temporal structure of trials in the experiment. Results of block-wise assessments using both analyses of variance (ANOVAs) and model comparisons are therefore only presented in the supplemental material (Fig. S1 and accompanying text; Table S3), for completeness and to allow for direct comparison with previous experiments. In the main text, we focus on a more fine-grained time course analysis that uses rolling regression.

For this rolling regression, a linear model with four *β*-coefficients (Intercept, FP, S1 type and their interaction) was defined on each trial, using data from a 60-trial window surrounding that trial. By means of a rolling window, trial-wise estimates of each *β*-coefficient were obtained, resulting in four *β*-time courses for each participant which expressed how each term developed throughout the experiment. For each coefficient, we subtracted the first value as a baseline, leading to a measure of how each coefficient evolved with respect to the start of the experiment (Δ*β*).

We then subjected these time courses to cluster-based permutation t-tests (Maris and Oostenveld, 2007), in order to identify whether behavior significantly changed over the course of the experiment while correcting for multiple comparisons in a manner that considers the temporal structure of the measurements. Clusters were identified as adjacent time points where a univariate test indicated that Δ*β* significantly (|*t*(48)| > 2.01) deviated from zero, that is, differed from the first measure in the time course. The sum of all *t*-values in a cluster was used as a test statistic. A *p*-value was determined for each cluster by testing the resulting value against a nonparametric null distribution of cluster *t*-values derived from 10,000 random permutations. Clusters were deemed significant at *α* = 0.05.

### Sample size and power

The effects of differential preparation for different S1 types are expected to gradually grow over the course of the experiment as participants learn. Therefore, it is difficult to evaluate statistical power, as it is contingent both on the unknown magnitude of the overall effect on preparation, as well as on how quickly this is acquired. As a heuristic to justify sample sizes, we ran power simulations using the ‘simr’ package (Brysbaert & Stevens, 2018; Green & MacLeod, 2016) with effect sizes and variances derived from earlier work (Los et al., 2021, Experiment ??). We isolated the data from the Transfer phase from that experiment and fit a full S1 type × FP model as defined above. Next, we scaled down the coefficients for the main effect ‘S1 type’ and for the S1 type × FP interaction effect. Then, we simulated 250 new ‘Transfer phase’ - results with different combinations of sample sizes (35–45 participants) and effect size scales (at 15–25% of the original effect sizes), and determined power as the proportion of simulations with significant S1 type effects from likelihood ratio tests.

Results indicated that with effect sizes at 20% of the original effect, a sample size of 45 participants yields a 97.6% power across the Transfer phase, and 79.2% if blocks are analyzed separately. Of note, with the same sample size, an effect size scaled down to 15% yielded a power of 85.2% across the Transfer phase, but only 48.00% for detecting effects in individual blocks. We chose to collect data from 50 participants, after which one was excluded due to poor performance as specified above.

## Results

### Questionnaire

For only four participants their response to the open question suggested they had been aware of the contingency. In the MC question, these participants correctly identified the predictive nature of S1. None of our statistical inferences regarding RT were any different upon excluding these participants, and therefore their data remained included for all analyses. Of the remaining 45 participants, 29 (= 64%) answered to the MC question that they did not know which image category had been paired with short or long FPs. Remaining responses (20 participants) did not significantly differ from chance accuracy (13 correct, *χ*^2^(1) = 0.95;*p* = 0.331). Thus, participants were generally unaware of the contingency.

### Preparation across phases

**Fig. 2:**
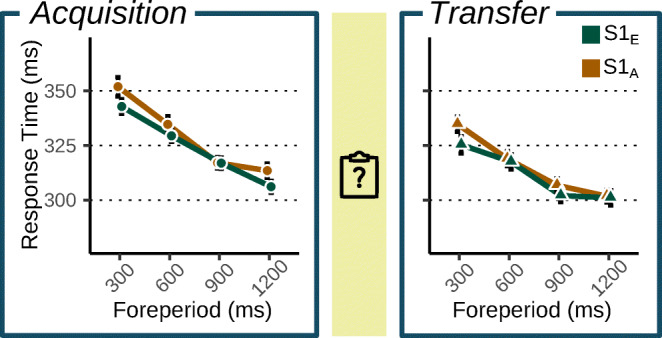
Mean response time per foreperiod in each phase. *Note*. Separately plotted for trials with different S1 types, during the Acquisition phase (left) and the Transfer phase (right). In both phases, the RT-FP curve is steeper for trials preceded by *S*1_*A*_ than *S*1_*E*_, suggesting differential preparation for different S1 types. Error bars indicate 95% Cousineau-Morey confidence intervals of within-subject effects (Cousineau, 2005; Morey, 2008)

Figure 2 depicts the RT-FP curve, separately for the different S1 types during the Acquisition and Transfer phase. While subtle, this curve is flatter for trials paired with *S*1_*E*_ types than *S*1_*A*_ types in both phases, indicating that the categories yielded differential preparation. LMM comparisons supported this observation: the best model expressed RT as a function of FP, S1 type and their interaction, with an additional main effect of Phase. This model was vastly preferred over a simpler model omitting ‘S1 type’ and the S1 type × FP interaction (Δ*BIC* = 39.28;*BF* > 1000). A main effect of ‘Phase’ was supported (Δ*BIC* = 419.92;*BF* > 1000), with moderate evidence against it interacting with FP (Δ*BIC* = − 3.02;1/*BF* = 4.52). A three-way interaction between these predictors was not supported (Δ*BIC* = − 15.27;1/*BF* > 1000).

Traditional ANOVA analyses generally supported these conclusions (Table S2 on OSF). All predictors had a significant main effect (*F*(1,48) >= 18.8;*p* < 0.001). Counter to the LMM conclusions, the ANOVA also revealed a significant FP × Phase interaction (*F*(1,48) = 18.97;*p* < 0.001), suggesting that there was a steeper RT-FP slope during Acquisition than during Transfer. Crucially, there was evidence for a two-way S1 type × FP interaction (*F*(1,48) = 6.49;*p* = 0.014), but not for a three way S1 type × Phase × FP interaction (*F*(1,48) = 0.68;*p* = 0.340).

We conclude that different S1 types led to differential preparation in both phases. These analyses gave no indication that this effect was attenuated in the Transfer phase, suggesting that the biased distributions in the Acquisition phase gave rise to long-lasting effects on preparation, persisting after the bias was removed and after participants were made aware that it no longer held. We next investigate the progression of this effect using the rolling regression analysis.

### Time course of differential preparation

**Fig. 3:**
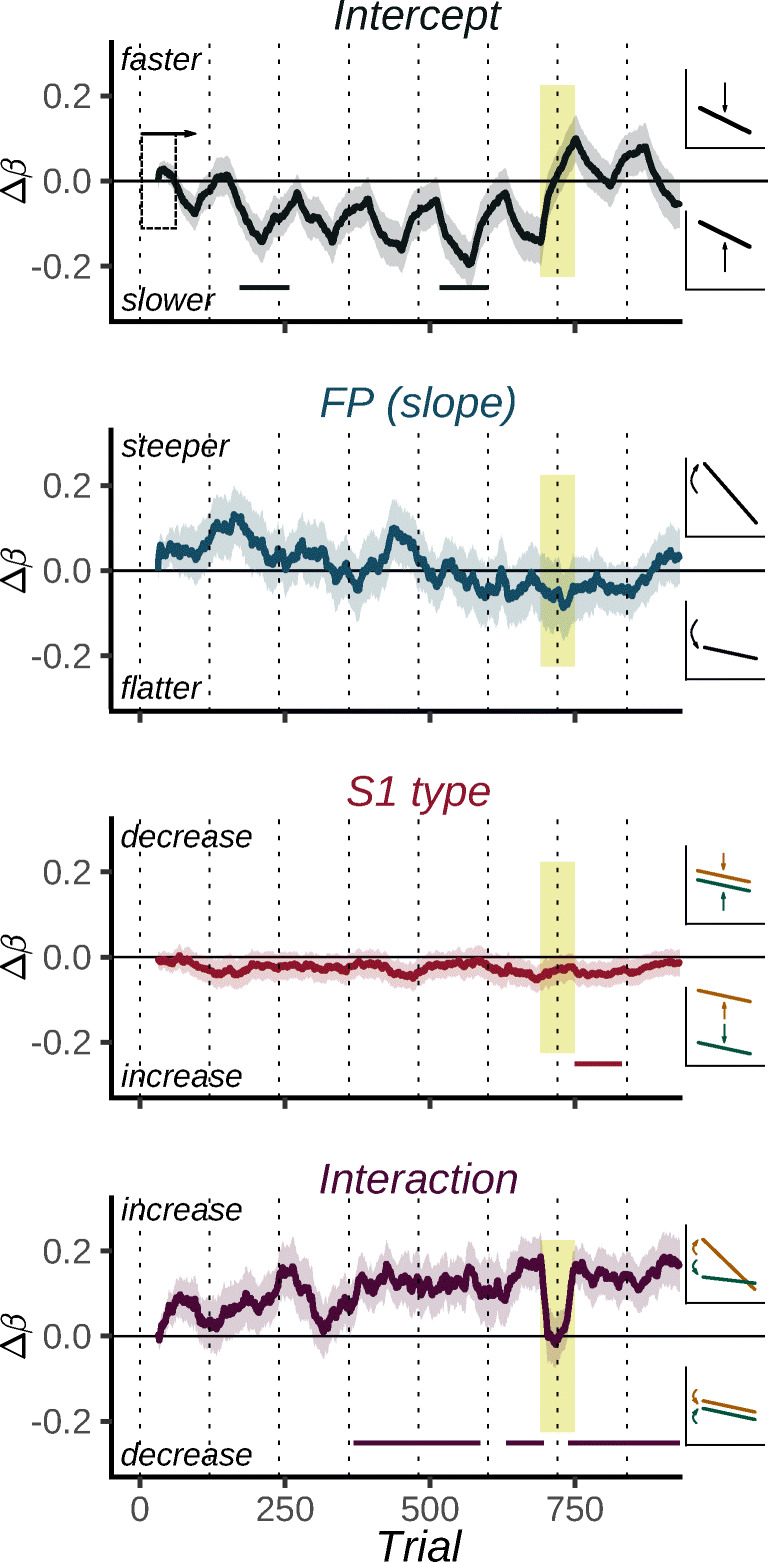
Development of model coefficients throughout the experiment. *Note*. Coefficients are determined using rolling regression. Δ*β* values are plotted at the center of the sliding window used to compute them (see dashed rectangle in the top panel). Therefore, there are no values at the start- and end of the experiment. Labels on the right illustrate how increases or decreases in Δ*β* affect RT-FP curves depicted in Fig. 2. Vertical dashed lines mark block breaks, and yellow shading indicates coefficients partially based on trials immediately following the questionnaire. Ribbons indicate within-subject 95% confidence intervals. Horizontal segments indicate clusters significantly different from baseline. Note that coefficients were determined from fits to inverse RT. The panels on the right indicate how positive and negative fluctuations are reflected in the RT-FP curve

Rolling regression yielded a per-participant time course of Δ*β* for each coefficient, which reveals how preparation changed since the start of the experiment. Mean time courses for each coefficient are plotted separately in Fig. 3. The time course of the Intercept (top row) shows that participants in general responded gradually slower throughout Blocks 1–6. This was accompanied by RT-fluctuations at a faster time-scale: In each block, participants became slower, then returned to baseline after each block break. This pattern suggests that participants might have gotten somewhat fatigued throughout each block, but that block breaks allowed them to largely recover to baseline. Furthermore, it suggests that the longer interruption between the Phases led to a more pronounced speeding up for the remaining two blocks. The sawtooth-like pattern was present in all blocks, but yielded only two significant clusters: one during Block 2 and one during Block 5.

Importantly, these Intercept-fluctuations were not reflected in other coefficients. Turning to the FP-coefficient, it seemed that compared to the start of the experiment, participants had somewhat steeper RT-FP curves in Blocks 1–4 and shallower curves in Blocks 6 and 7. However, these fluctuations were marginal, and were not reflected in significant clusters. Therefore, we conclude that overall temporal preparation remained largely consistent throughout the experiment. The main effect of S1 type was also found to be relatively stable; with respect to its initial value, Δ*β* displayed a slight increase in this effect, meaning that participants responded somewhat faster on *S*1_*E*_ trials than on *S*1_*A*_ trials. Although this tendency qualitatively developed early in the Acquisition phase, it came to expression in only one significant cluster which coincided with Block 7.

Most notable, however, was the development of the S1 type × FP interaction coefficient. Throughout Blocks 1–6, this coefficient displayed a gradual increase, reaching a maximum value during Block 6. This is expressed by two significant clusters spanning trials in Blocks 4 – 6, and shows that differential preparation for the two S1 types indeed gradually became more pronounced during the Acquisition phase. Interestingly, starting with the first trials of Block 7 immediately following the questionnaire, a sharp decrease in this coefficient is observed; that is, the effect almost instantly reverts to baseline level. Likely, this drop results directly from the information provided after the questionnaire: participants were informed that the cues had no predictive value, and may have actively suppressed or counteracted any associative guidance. However, within the same block, differential preparation returned to the level it had before the questionnaire. This was reflected in a third significant cluster that spanned most of Blocks 7 and 8.

Note that the time course of these effects, including the duration of the sudden ‘drop’ of the interaction coefficient, is determined not only by the time course of behavioral changes, but also affected by the window size chosen for the rolling regression analysis. This reflects a trade-off between the temporal resolution and the accuracy of coefficient estimates. In Fig. S2 on OSF we therefore present results of analyses with different window sizes (40 and 120 trials). These generally followed the same pattern as the analyses presented here. Notably, the results with a shorter window of 40 trials highlight that the ‘dip’ in the S1 type × FP time course at the start of the Transfer phase was very short-lived. The brevity of this effect could therefore explain why our earlier work, using block-wise analyses, consistently led us to conclude that this transition between phases had no noticeable effect on differential preparation (Los et al., 2021; Los et al., 2017; Mattiesing et al., 2017).

To assess whether this pattern was also present in earlier experiments using only two S1s instead of unique instances of two categories, we present an exploratory re-analysis of three experiments from (Los et al., 2021) using rolling regression (Fig. S3 on OSF). This concerns two experiments where S1 pairs gave rise to differential preparation, and one where the S1 pair did not. The time course of the S1 type × FP interaction in these experiments was qualitatively similar that in Fig. 3. Differential preparation gradually developed during the Acquisition phase, followed by a ‘dip’ at the start of the Transfer phase following explicit information. Following this dip, differential preparation recovered almost immediately to the level it had at the end of Acquisition, and subsequently attenuated only minimally throughout the Transfer phase. Interestingly, a qualitatively similar dip was found in the experiment where S1s did not yield differential preparation. This supports an interpretation of this dip as reflecting a short-lived intentional change in preparation that is generally independent from the slow modulations that engender from associative learning.

## Discussion

Recent research has demonstrated that temporal preparation is guided by long-lasting associations in memory, either by relating unique images to specific FPs (Cravo et al., 2017), or by statistically pairing stimuli with different FP distributions (Los et al., 2021). The present study illustrates associative guidance generalizing to novel stimuli: By pairing unique photographs of faces and scenes with different FP distributions, we found that participants adjusted their temporal preparation in response to never-before seen photographs of either category. In a subsequent Transfer phase, where the category-FP contingency was removed, we found that differential preparation to novel stimuli of either category nevertheless persisted.

Our results suggest that such differential preparation does not rely on strategic, voluntary control. When asked, participants seemed generally unaware of the contingency, even though image categories triggered differential preparation according to their associated FP distribution. Furthermore, participants were still guided by past associations in the Transfer phase, even though they had been informed that the category-FP contingency no longer applied (cf. Los et al., 2021). This aspect contrasts with the work of Cravo et al., (2017), where participants were explicitly instructed to learn and utilize the pairing of individual images with their associated FP. Our findings raise the possibility that participants in that study might have demonstrated similar memory-guided preparation even if they would have been unaware of the image-FP pairings. This possibility is supported by analogous findings on implicit *spatial* cueing by individual scenes and search displays (Brockmole and Henderson, 2006; Chun & Jiang, 1998).

While our results demonstrate that countermanding information does not override effects of implicit associations (cf. Los et al., 2021), our rolling regression analyses revealed a striking nuance to this claim. That is, differential preparation was briefly abolished following this information but was promptly reinstated, a pattern also found in earlier, similar experiments (Fig. S3 on OSF). While we cannot verify empirically what caused this disruption, this effect notably manifested in the *S*1 × *FP* coefficient while leaving overall preparatory behavior largely unaffected. This might therefore reflect participants intentionally using the new information about distributions in the Transfer block, in a manner that counteracted the effect of instructions. However, the observation that differential preparation swiftly recovered suggests that preparation may be ‘by default’ guided by associations. Together with earlier work outside of the context of preparation, these results outline a complex interplay between implicit associative guidance and guidance by explicit awareness. For example, research on spatial attention has put forward the hypothesis that attentional selection of statistically regular features are imperative for implicitly learning these regularities (Turk-Browne et al., 2005; Jiang & Chun, 2001), and associations can thereby be shaped by explicit control. Recent work on Stimulus-Response bindings has similarly illustrated how instructions can shape the nature of categorical associations (Longman et al.,, 2018; Longman, Liefooghe, & Verbruggen, 2019; Waszak, Wenke, & Brass, 2008). Conversely, research on feature-based attention suggests that instructions are ineffective at overriding previously acquired implicit guidance (Kruijne & Meeter, 2016; Leber & Egeth, 2006), much like the current results. Taken together, these findings suggest that instructions can have a formative role in the development of new implicit associations, but that once associations are formed, explicit strategic control is only marginally able to counteract them (see also Feldmann-Wüstefeld, Uengoer, & Schubö, 2015).

The main purpose of the rolling regression was to offer fine-grained insights into how differential preparation developed during Acquisition and Transfer. The analysis revealed a gradual development of this effect, peaking at the end of Acquisition. Of note, this smooth increase reflects the development of differential preparation collapsed across participants (see Spaak & Lange 2020, for a consideration at the individual level). Compared to previous experiments (Fig. S3 on OSF), associative guidance was relatively small and took long to acquire, likely due to the heterogeneity of S1s (cf. Feldmann-Wüstefeld & Schubö, 2014). Nevertheless, differential preparation was robust once acquired, persisting well into the Transfer phase despite the change in underlying FP distributions. In other experiments, with longer Transfer phases, we similarly observed that the S1 type × FP interaction barely attenuated across Transfer blocks.

This fine-grained characterization of the development and persistence of memory-guided effects can help constrain models of temporal preparation. The gradual acquisition of differential preparation and its longevity throughout the Transfer phase illustrate how temporal preparation is affected by long-term memory and sluggishly adapts to changing environmental statistics (see also Crowe & Kent, 2019; Crowe, Los, Schindler, & Kent, 2021; Los et al., 2017; Mattiesing et al., 2017; Visalli et al., 2019; Visalli, Capizzi, Ambrosini, Kopp, & Vallesi, 2021). Many probability-driven models characterize preparation as guided by static representations of the current FP distribution (Janssen & Shadlen, 2005; Grabenhorst et al., 2019; Trillenberg et al., 2000; Vangkilde et al., 2013), foregoing the role of memory and learning. Transfer effects like those in the present study illustrate the need for a flexible basis for preparation, subject to learning and updating (e.g., de Jong, Akyürek, & van Rijn, 2021; Meindertsma et al., 2018; Vissali et al., 2019), just as in MTP (Los et al., 2014).

Critically, we demonstrated differential preparation generalized to novel stimuli, an aspect of memory and learning previously not considered in preparation studies. Such generalization may be a consequence of the accumulated contribution of individual stimuli of a different category, or could result from direct associations at the category level. Different instance theories offer mechanistic accounts of how such generalization could arise (Hintzman, 1986; Schapiro, Turk-Browne, Botvinick, & Norman, 2017; Kumaran & McClelland, 2012; Altmann, 2017), and based on their similarity to MTP we predicted similar generalization to arise within the context of the FP paradigm. In MTP, preparation results from Hebbian associations between a dynamic representation of time that is elicited by the S1, and processes of motor activation and inhibition. Our results may be explained within this framework by assuming a degree of overlap between the representations elicited by S1s of the same category. The ability to generate differentiating predictions with different S1s would then be defined by the distinctiveness of these representations. This would fit with the observation that differential preparation effects were stronger and quicker to develop with highly distinctive, cross-modal S1 pairs (Los et al., 2021).

Taken together, these results demonstrate that associations formed during temporal preparation can yield predictions for novel stimuli of the same category. Additionally, they provide further evidence that rather than explicit instructions, it is primarily practice that makes preparation perfect.

## Electronic supplementary material

Below is the link to the electronic supplementary material.

(PDF 3.23 MB)

## Footnotes

1 A heuristic for familiarity was derived from Google Trends statistics queried on the name of the image subject.
