# Supplementary material for: Implicitly learning when to be ready: From instances to categories
Source: Psychon Bull Rev. 2021 Oct 28;29(2):552–62. doi: 10.3758/s13423-021-02004-w (PMC9038822; doi:10.3758/s13423-021-02004-w)
Supplement: Supplementary file 1 — (PDF 3.23 MB) [file 13423_2021_2004_MOESM1_ESM.pdf]

Implicitly learning when to be ready: from instances to categories
Supplemental Material

Wouter Kruijne[1], Riccardo M. Galli[2], and Sander A. Los[3]
[1]University of Groningen
[2]UKE Hamburg
[3]VU Amsterdam

Wouter Kruijne https://orcid.org/0000-0001-6358-7468, Riccardo M. Galli, Sander A. Los https://orcid.org/0000-0003-3954-4817
WK is supported by VICI grant 453-16-005 from the Netherlands Organisation for Scientific research (NWO) awarded to Hedderik van Rijn. Correspondence concerning this work should be addressed to: Wouter Kruijne, Department of Experimental Psychology, Faculty of Behavioral and Social Sciences, University of Groningen, Grote Kruisstraat 2/1, 9712 TS Groningen.
E-mail: w.kruijne@rug.nl

Table S1

*Participants were first asked an open question (*did you notice anything about the experiment? Were there any oddities, regularities, repetitions, etc. that you noticed?*). In a subsequent MC question they were asked to guess which category they believed predicted mainly short/long FPs, or to indicate they did not know (blank cells). Responses and MC accuracy (first and last column) are sorted on the nature of the $S1_A$ (**F**aces or **S**cenes). Boldface indicates participants who were considered 'aware' of the contingency.*

Response to open question	$S1_A$	MC Correct?
• Often pictures of prisoners	F	
• I did not find a pattern	F	
• In each block there was the circle about 5 times in a row on the left and then about five times in a row on the right with really short interval between picture and circle.	F	
• I did not notice anything, one time I thought a landscape picture was in there twice but I do not know whether that is true.	F	
• The pictures showed either different people, landscapes or buildings.	F	
• No oddities regularities or repetitions I think	F	
• To my recollection, there was no repetition, each picture was unique	F	
• Some pictures had black outlines around them	F	
• No I didn't notice anything	F	
• In the experiment you get to see pictures of faces and scenes, I think there is no specific relation between the pictures of the faces and the pictures of the scenes if we look at the location where the dot appears	F	
• The pictures are mostly of people and landscapes.	F	
• A lot of political persons	F	
• Many faces sometimes expressing emotions (sad, happy). a lot of buildings (houses, farms, schools) and also nature (forest, river)	F	
• Repetition of pictures and same kind of landscapes/objects but different picture	F	
• Sometimes the circle appears faster and sometimes slower, also many of the stimuli are similar, faces without body, nature etc.	F	
• I can kind of guess the pattern. in the beginning of each block, left and right will keep switching for 4 to 6 times (the repetition will not be more than 3). Afterward, left is more likely to keep the repetition for 4–8 times, then switch back to right. The repetition of right will not be more than 6 (around 3 to 6 I think).	F	
• Regularities	F	0
• I did not notice anything, besides the regular themes of pictures (people, landscapes).	F	0
• I did not	F	0
• Sometimes there were series on the right or left, and then suddenly the opposite.	F	0
• I did notice some repetitions regarding the pictures; some appeared a couple of times and some others just one in one block	F	1
• I did not notice anything odd about the experiment	F	1
• I did not	F	1
• No I did not.	F	1
• **I noticed that the circle appeared faster, when objects instead of faces were shown**	F	1
• Pictures of faces and random things are presented	S	
• I did not notice anything special, only that sometimes it takes a bit longer before the dot appear.	S	
• Pictures were often faces and especially often faces of celebrities	S	
• No	S	
• No, I did not notice any oddities, regularities or repetitions.	S	
• I was purely focusing on the placement of the dots, not on the images itself.	S	
• I did not notice anything	S	
• I noticed that I made like 3 mistakes that were not detected by the program. When I by accident already pressed Z/M before the circle was shown.	S	
• The direction of the gaze of a person seems to play a role / the attentional center of a picture	S	
• A lot of dots on the left. Seemed like some political figures. For the rest, not much.	S	
• I did not	S	
• I did not	S	
• I didn't notice anything	S	0
• I noticed that there were neutral pictures and pictures that try to lead you to a certain side (left or right).	S	0
• I did not find a pattern. the target dot seemed to be placed closer or farther from the fixation dot.	S	0
• Circles from time to time where either closer or more far away from the center.	S	1
• There were portraits of people with the same background – probably school photos.	S	1
• No	S	1
• There were a lot of faces, and then (parts of) buildings. And sometimes the circle appeared later than other times.	S	1
• No I did not notice any regularities or etc	S	1
• The pictures weren't very different from one another, mainly stayed within a few categories, for example: people, nature, etc.	S	1
• **I think I was responding faster after I saw a face than after I saw another scene.**	S	1
• **Longer gap after images of landscapes, houses, etc. than after images of people/ faces**	S	1
• **I expected to be faster when I saw faces than when I saw objects**	S	1

Figure S1

Average response time per foreperiod in each block

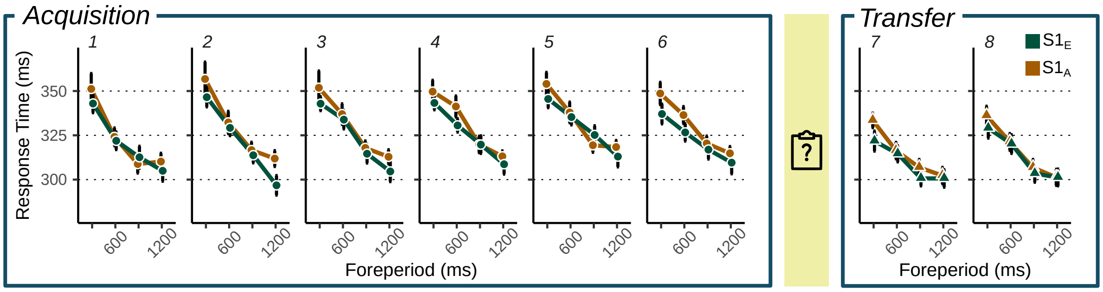

Note. Separated for trials with different S1 types. The differences in preparation for different S1 types are most pronounced at the end of the Acquisition phase, and persists in the Transfer phase. Error bars indicate 95% Cousineau-Morey confidence intervals of within-subject effects (Cousineau, 2005; Morey, 2008).

In the main text, we present analyses of RTs in either phase, collapsed across blocks (Figure 2; see also Table S2). Here, we present a block-wise analysis, intended to allow for direct comparison with our earlier work (Los et al., 2021). Like in the main text, we stress that this analysis treats blocks as independent, which greatly reduces the statistical power given the relatively small effect size in the present experiment.

The RT-FP curves in different blocks are depicted in Figure S1. They reveal an overall trend in the development of associative preparation: while in Block 1, there is no indication that different S1s led to different behavior, a pronounced difference can be observed in Block 6, in particular with shorter FPs. Block-wise model comparisons generally supported the FP-only model for Blocks 1–5 ($-14.9 < \Delta BIC < 0.46; 0.79 < 1/BF < 1723.56$). However, for Block 6, the model including the interaction was strongly preferred ($\Delta BIC = 5.05; BF = 12.50$). After participants had been informed that the contingency no longer held, there was still strong evidence supporting differential preparation in Block 7 ($\Delta BIC = 13.22; BF = 743.13$). Thus, results indicate that differential preparation developed throughout the Acquisition phase and persisted in the Transfer phase. In Block 8, the interaction-model was no longer supported ($\Delta BIC = -8.25; 1/BF = 62.10$).

The block-wise ANOVA (Table S3) generally supported these inferences, although the S1 type × FP interaction was only significant in Block 7. Nevertheless, we found that the effect size (η_p^2) of this interaction consistently increased in each subsequent block up to Block 7, only to decrease again in Block 8. This points to a consistent, gradual development of differential preparation throughout the Acquisition phase, which might go undetected in block-wise analyses due to a lack of power. The rolling regression analysis allows for a more fine-grained evaluation of the gradual development of this effect.

Table S2

ANOVA table for the S1 type × Phase × FP analysis on average RT per condition. Significant effects are marked by p-values in boldface.

			df	*SSQ*	*MSQ*	*F*	*p*	η_p^2
FP			1	127004.20	127004.20	189.3	< **0.001**	0.80
			48	32197.88	670.79			
S1 type			1	4467.50	4467.50	20.9	< **0.001**	0.30
			48	10240.50	213.34			
Phase			1	33415.45	33415.45	18.9	< **0.001**	0.28
			48	85055.68	1771.99			
S1 type	× FP		1	671.82	671.82	6.5	**0.014**	0.12
			48	4965.50	103.45			
Phase	× FP		1	1936.24	1936.24	19.0	< **0.001**	0.28
			48	4898.20	102.05			
S1 type	× Phase		1	92.93	92.93	0.8	0.381	0.02
			48	5697.05	118.69			
S1 type	× Phase	× FP	1	96.14	96.14	0.9	0.359	0.02
			48	5390.65	112.31			

Table S3

Independent ANOVA, per block, on average RT. Bold p-values are significant at $\alpha = 0.05$ (without multiple comparisons correction).

	FP			S1 type			FP × S1 type		
Block	*F*	*p*	η_p^2	*F*	*p*	η_p^2	*F*	*p*	η_p^2
1	172.6	< **0.001**	0.78	8.0	**0.007**	0.14	<0.1	0.919	<0.01
2	163.7	< **0.001**	0.77	13.8	**0.001**	0.22	0.2	0.667	<0.01
3	121.1	< **0.001**	0.72	9.8	**0.003**	0.17	0.2	0.626	<0.01
4	141.9	< **0.001**	0.75	7.6	**0.008**	0.14	1.4	0.239	0.03
5	130.3	< **0.001**	0.73	11.8	**0.001**	0.20	1.8	0.181	0.04
6	96.5	< **0.001**	0.67	19.4	< **0.001**	0.29	1.9	0.174	0.04
7	131.9	< **0.001**	0.73	8.8	**0.005**	0.15	5.9	**0.019**	0.11
8	111.7	< **0.001**	0.70	2.4	0.129	0.05	2.1	0.151	0.04

Figure S2

Rolling regression analysis with different window sizes than 60 trials (main text)

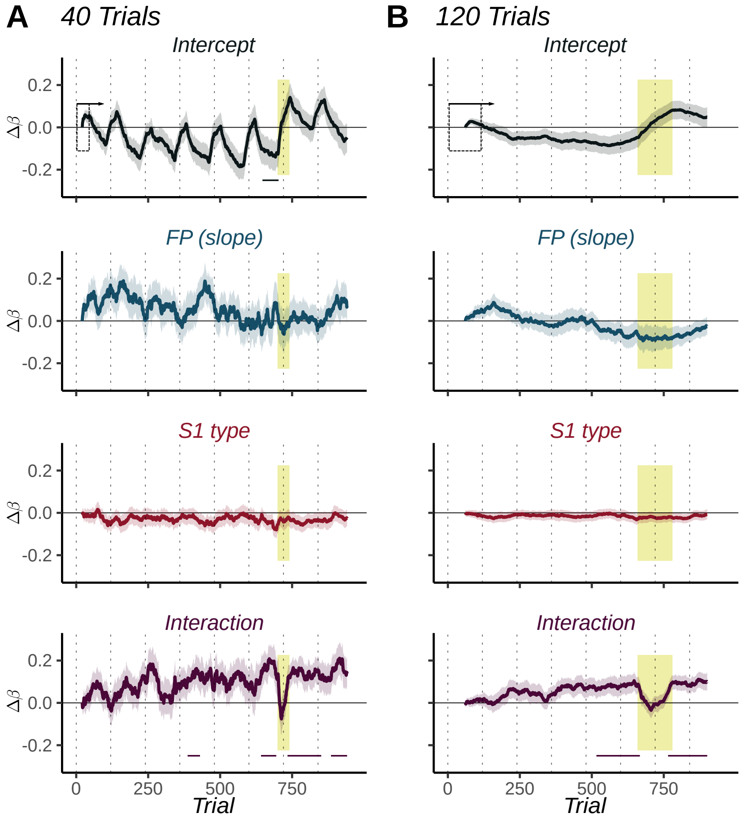

Note. **A** A smaller window of 40 trials. **B** A larger window size of 120 trials (the number of trials in one block). Window size affects the time scale of uncovered trends and effects (e.g. for the Intercept-term). Across all window sizes, however, the interaction effect (bottom graphs) gradually grows throughout the experiment, accompanied by a brief 'dip' after participants had been instructed that the S1-FP contingency no longer holds.

Figure S3

Rolling regression applied to experiments from Los et al. (2021)

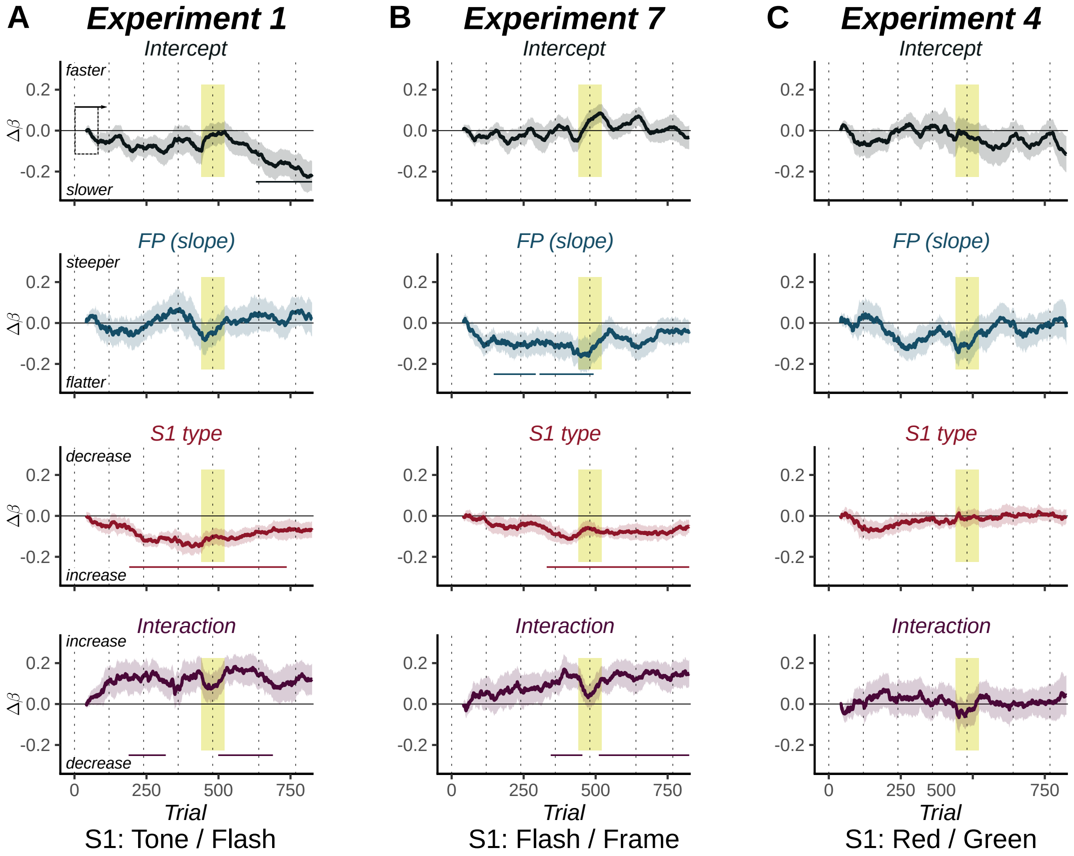

Note. Experiments were similar to that in the main text, but used pairs of homogeneous, repeating S1s. Different experiments tested different S1 pairs used as $S1_E$ and $S1_A$ **A** Experiment 1 used a highly dissimilar 'flash' and 'tone' as S1s, and showed pronounced differential preparation. **B** Experiment 7 used unimodal S1s, a central flash or a peripheral frame. **C** Experiment 4 used a red and green S1s, and their predictive quality was explained beforehand. Nevertheless, this pair did not give rise to differential preparation. (See text below for more detail.)

We reanalyzed data from previous experiments (Los et al., 2021) using rolling regression, for two main reasons. First, these analyses allow for direct comparison to the present results (main text Figure 3; Figure S2). Second, we sought to determine whether the 'dip' in differential preparation following instructions had also been concealed in these data. We analyzed data from three selected Experiments: (A) In Experiment 1, S1s were either a filled central circle or a 1000Hz tone, each presented for 50ms. Block-wise analyses revealed pronounced differential preparation effects, significant in all individual blocks. (B) In Experiment 7, S1s were either a filled circle (as Experiment 1) or a large, rectangular frame. These S1s, with the same modality, still produced differential preparation, but developing slower and less pronounced. (C) In Experiment 4, S1s were red or green central filled circles (50ms). Participants were additionally informed of the contingency before the start of

the experiment, but did not display differential preparation. These experiments employed virtually the same design as the present experiment, but had a shorter Acquisition phase (480 trials), and longer Transfer phase (384 trials). Of note, they had smaller sample sizes (N=20), and we therefore we used a larger window size (80 trials) for the rolling regression to maintain a sensible trade-off between temporal resolution and coefficient estimation. A general interpretation of the results is provided in the main text, focusing on differential preparation and the dip. Below, we describe the results for each coefficient in more detail.

Experiment 1: Tone vs. Flash. Just as in the main experiment, the Intercept in this previous experiment fluctuated with a rhythm that was in phase with block breaks, suggesting that participants developed a degree of fatigue or boredom but recovered to baseline performance immediately after a break. Remarkably, near the end of the experiment, block breaks seemed less effective in bringing performance back to baseline in this sample. Overall RTs increased, resulting in a significant deviation marked by a cluster in the last block. The main effect of FP, however, did not follow this pattern, which suggests that temporal preparation overall might not have been affected by these 'fatigue'-effects.

Coefficients for 'S1 type' and 'S1 type × FP' are clearly indicative of differential preparation, marked by significant clusters in both Acquisition- and Transfer phases. Note that differential preparation already developed early in the Experiment, as was also indicated in the block-wise analysis. This rapid development actually may have lead to an underestimation of differential preparation throughout the curve as a whole: $\Delta\beta$ is computed with respect to a baseline estimated from the first 80 trials, which presumably already contained a degree of differential preparation. Future work can overcome this issue by incorporating an 'unbiased' first block, ensuring accurate estimates of the baseline.

Following the 'dip', differential preparation effects were somewhat attenuated during the Transfer phase, albeit much slower than the rate at which they were acquired.

Experiment 7: Flash and Frame. Again, we found fluctuations in the Intercept term, aligned to the block breaks. Nevertheless, performance was relatively stable in this experiment and no significant deviations were found. Interestingly, we did find a significant deviation for the FP slope-coefficient in this experiment. It suggests that participants showed relatively flatter preparation curves in the majority of the experiment, compared to their behavior in the first block. This could reflect a practice effect, as it effectively means that participants were overall better prepared when responding to shorter intervals.

Compared to Experiment 1, differential preparation developed more slowly, and clearly only peaked at the end of the Acquisition phase. It was again interrupted by a 'dip' at the start of the transfer phase. but subsequently persisted throughout the Transfer phase. Both for the main effect of S1 type and the S1 type × FP interaction coefficient, the corresponding significant cluster extended until the end of the experiment.

Experiment 4: Red and Green. One of the more surprising findings of Experiment 4 is that even though participants were instructed beforehand of the predictive value of red and green S1 cues, they did not seem to translate this information into differential preparation. The rolling regression analysis also gave no indication of differential preparation, with no significant clusters for any of the coefficients. Nevertheless, the results did reveal a qualitative 'dip' in the interaction-coefficient at the start of the Transfer phase. This may suggest that the dip reflects a strategic, voluntary response that is independent of whether any associations are formed that give rise to differential preparation.
